# Wound Management Amongst Doctors in Training: A Cross‐Sectional Study of Education and Capability

**DOI:** 10.1111/iwj.70674

**Published:** 2025-05-08

**Authors:** Hamza Duffaydar, Octavi Casals‐Farre, Jessica Morgan, Harri Jones, Hassan Duffaydar, Amy Smith, Charles Kimberly, James Brock, Rhidian Morgan‐Jones, Arwel Poacher

**Affiliations:** ^1^ Core Surgical Trainee, Department of Oral and Maxillofacial Surgery Cardiff and Vale University Health Board Cardiff UK; ^2^ Foundation Year 2 Doctor, Department of Critical Care Cardiff and Vale University Health Board Cardiff UK; ^3^ Foundation Year 2 Doctor, Department of Surgery Buckinghamshire Healthcare NHS Trust Buckinghamshire UK; ^4^ Foundation Year 2 Doctor, Department of Trauma and Orthopaedics Cardiff and Vale University Health Board Cardiff UK; ^5^ Machine Learning (MRes) Student Imperial College London London UK; ^6^ Orthopaedic Trainee, Trauma Department Cardiff and Vale University Health Board Cardiff UK; ^7^ Consultant Trauma and Orthopaedic Surgeon Cardiff and Vale University Health Board Cardiff UK; ^8^ Welsh Clinical Academic Track, Department of Biomedical Sciences Cardiff University Cardiff UK

**Keywords:** Doctors in Training, Education, Medical School, Wound Healing, Wound Management

## Abstract

Wound care in the UK is a resource‐intensive challenge, costing the NHS £8.3 billion annually and growing with an ageing population. However, there is no evidence of whether doctors in training receive adequate teaching to perform wound care competently. Our study aimed to investigate doctors’ confidence when assessing and managing wounds and their preferred learning modality. This cross‐sectional study comprised 262 doctors training across the UK. We assessed the correlation between confidence in managing wounds, seniority in training, and trainee speciality. Only 65% of doctors had received teaching on wound healing during medical school, and 25% received further teaching during postgraduate training. Surgical trainees felt more confident in assessing and managing wounds than their medical counterparts (*p* < 0.01), and surgeons were the only group demonstrating a positive correlation between seniority and confidence in wound management (*p* = 0.02). All speciality groups favoured bedside teaching and thought wound management was integral to clinical practice. Our study has shown that training is sub optimally delivered and insufficient for trainee requirements. Incorporating dedicated teaching across specialities will be essential to manage the increasing demand for wound care.

1

**Summary**

- Wound care education is insufficient across UK medical training: Only 65% of doctors received wound care teaching in medical school, and just 25% during postgraduate training, leaving many trainees underprepared.
- Lectures dominate but are least preferred: While most undergraduate teaching is lecture‐based (75%), trainees strongly preferred hands‐on, clinical or simulation‐based learning.
- Surgical trainees show the highest confidence: only surgical trainees demonstrated increased confidence in wound assessment and management as their training progressed GP and medical speciality doctors did not.
- All value wound care, but skills are lacking: Regardless of speciality, all trainee groups believed wound care is integral to future practice, yet reported poor confidence and a desire for more practical teaching.
- Urgent need for structured, cross‐speciality training: The study recommends widespread reform in wound care education to improve clinical competence and meet growing patient needs, especially as GPS take on more wound management roles.

## 2 Introduction

Wound management in the UK is a resource‐intensive challenge, with one in fifty people relying on wound care services for chronic lesions [1]. The economic burden of wound care to the National Health Service (NHS) was estimated at £8.3 billion in 2018, with costs growing alongside an ageing population [2, 3]. Chronic wounds also have a significant psychological and physical impact on patients' lives [4]. While the care of individuals with complex wounds is primarily handled in the community by district nurses and General Practitioners (GPs), hospital doctors are more likely to deal with acute wounds [5, 6]. Therefore, the education of medical practitioners across all grades and specialities is paramount due to the high involvement in wound care throughout their careers [3].

The National Wound Care Strategy Programme has made strides in providing resources and frameworks, such as the Core Capabilities Framework, which sets out the knowledge and skills needed for wound care [7]. This framework is available for a broad healthcare audience, though it lacks a focus on physician‐specific competencies and wound management training. The programme also provides comprehensive management recommendations and clinical pathways. This gap highlights a need for improved access to targeted wound care training for doctors in the UK to build a more uniform competency level across the healthcare profession.

Previous studies have shown that medical students receive limited teaching on wound management and that foundation doctors lack the knowledge and confidence to manage wounds. However, no study to date has compared the confidence of doctors in training in assessing and managing wounds amongst different specialties [8, 9, 10, 11, 12]. There is also no study exploring the relationship between progression in medical training and confidence in wound management.

The primary aim of the study was to assess confidence in assessing and managing wounds clinically between different trainee groups: foundation, medical, general practice (GP) and surgical trainees. The secondary aim was to investigate the relationship between seniority in training and confidence in managing wounds. We also sought to understand what proportion of doctors in training received teaching on wound healing.

## 3 Methods

The authors designed a questionnaire with 20 questions using the CHERRIES checklist [13]. The questionnaire primarily consisted of a combination of Yes/No, multiple choice and rating (Likert 10‐point scale) questions. The questionnaire also included questions where participants were invited to respond in free text. This was used to answer open questions, for example, what they would like to be taught with regard to wound healing. Different styles of question were tailored to the expected variety in answers. The questionnaire was created and distributed as a Google Form (Google LLC USA) and did not require any participant‐identifiable details so that the results could remain anonymous. Before completing the form, participants agreed to have their responses used for medical research. The questionnaire was open to responses from doctors in training across the UK between March and September 2024. Only doctors who were currently in training and had attended a UK medical school were eligible to participate.

Data analysis was performed using Python and Microsoft Excel by a statistician. Graphs and images were generated using Microsoft Excel. The Analysis of Variance (ANOVA) test was used for variables that followed a parametric distribution and the Kruskal–Wallis test was used for those that did not follow a parametric distribution. The Pearson Correlation test was also used to compare variables. Statistical significance was defined as a *p* value less than 0.05.

## 4 Results

**FIGURE 1:**
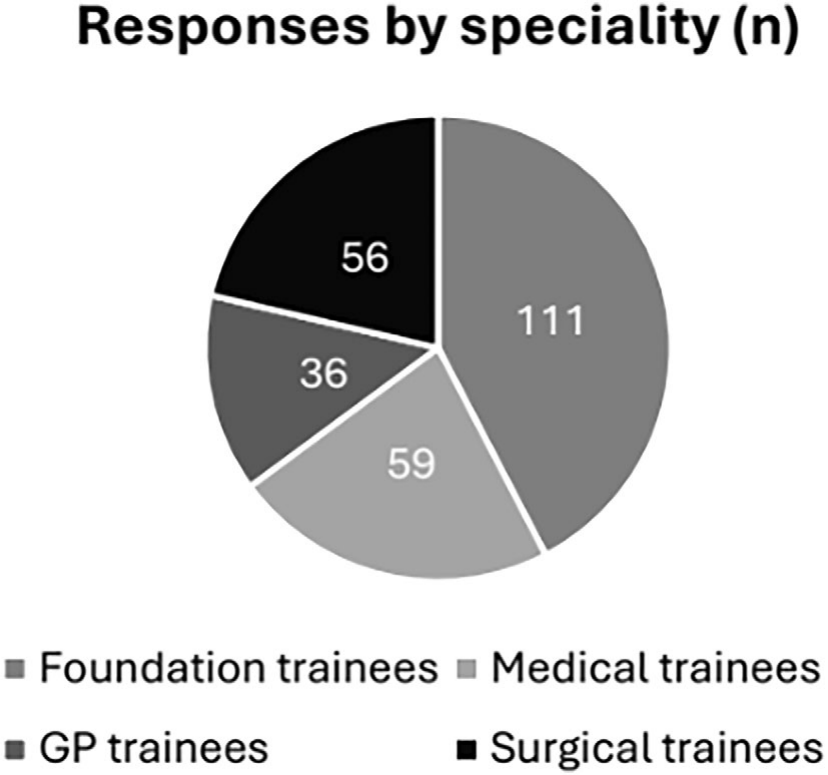
A Pie chart illustrating the characteristics of the study population according to speciality of medical training.

A total of 262 doctors in training responded to the questionnaire. The respondents included 111 foundation trainees, of which 53 were foundation year 1 doctors, 45 were foundation year 2 doctors and 13 were foundation year 3 doctors or junior clinical fellows. There were 34 responses from Internal Medical Trainees (IMT) and 30 responses from Core Surgical Trainees (CST). A total of 36 GP registrars, 25 medical registrars and 26 surgical registrars responded. Overall, Foundation trainees constituted 42% of respondents, medical trainees 23%, GP trainees 14% and Surgical trainees 21%. Figure 1 illustrates the total number of trainees as per their specialty.

## 5 Wound Healing Education at Medical School

Overall, 81% (212/262) of respondents reported that wound healing was included in the core medical curriculum. However, only 64.5% (169/262) of respondents received teaching on wound healing during medical school. For the remainder, the teaching was in the form of optional modules, electives or student‐selected components.

The most common format for wound care education in medical school was lecture‐based (75%), followed by simulated skills training (9%) and clinical workspace learning (9%). A combination of lectures and simulation was employed in 7% of cases. When asked about their preferred method for wound healing education, participants indicated a preference for clinical workspace learning (41%). Overall, 32% of respondents favoured a blend of lectures and simulation, while 19% preferred simulated clinical skills alone. Lecture‐based teaching alone was the least popular choice, selected by just 8% of respondents.

When doctors were asked to rate on a Likert scale of 1 to 10 if they received adequate undergraduate wound healing teaching to prepare them for their clinical practice, the mean score was 3.4, with a standard deviation (SD) of 1.7.

## 6 Wound Healing Education at the Postgraduate Level

We received responses from trainees who were currently in, or had completed, Foundation training from all 20 deaneries in the United Kingdom. Only 18% of participants received teaching on wound healing as part of their Foundation training. For those who did receive teaching during foundation training, the most popular teaching method was learning in the clinical workspace (54%), followed by lecture‐based teaching (35%). At the postgraduate level, which would include foundation training and further speciality training, the proportion of participants who received teaching in wound healing during their training rose to 25%.

When participants were asked what they had been taught about wound healing at the postgraduate level, the main themes were ‘healing by primary v/s secondary intention,’ ‘teaching on burns and open fractures’ and ‘stages of wound healing.’ Many participants also reported that they had learnt about wound healing in preparation for the Membership of the Royal College of Surgeons (MRCS) exam. When asked what topics they would like to be taught, responses included ‘assessment and management of chronic wounds’, ‘common dressings and their indication’, ‘different closure methods and when to use steri‐strips or glue or suture’, ‘phases of wound healing’ and ‘when to remove clips or non‐dissolvable sutures’.

## 7 Confidence Assessing and Managing Wounds

Table 1 summarises the participants' confidence in assessing wounds clinically, managing acute and chronic wounds, prescribing wound care products and the importance of wound care in their current and future clinical practice. Surgical trainees felt more confident in assessing and managing wounds alongside prescribing wound care products. They also felt that wound care was more relevant to their current clinical practice as compared to foundation, medical and GP trainees. The results were statistically significant (*p* < 0.01) when calculated using the ANOVA test. However, when asked if wound care would be important in their future clinical practice, all groups deemed wound care to be equally important, and there was no significant difference (*p* = 0.52) in their responses.

**TABLE 1:** Participant reported outcomes with regards to wound management.

Outcome: mean (SD)	Foundation trainees	Medical trainees	GP trainees	Surgical trainees	*p*
Confidence assessing wounds clinically	4.4 (2.2)	4.4 (1.6)	4.4 (2.1)	7.1 (2.2)	< 0.01^a^
Confidence managing acute & chronic wounds	3.5 (1.9)	4.0 (1.7)	4.0 (2.1)	6.9 (2.4)	< 0.01^a^
Confidence prescribing wound care products	2.6 (1.8)	3.2 (1.8)	3.5 (2.2)	5.4 (2.6)	< 0.01^a^
Importance of wound care in current clinical practice	6.2 (2.3)	6.7 (1.2)	7.9 (1.3)	8.0 (2.3)	< 0.01^a^
Importance of wound care in future clinical practice	7.5 (2.1)	7.3 (1.2)	7.9 (1.3)	7.6 (2.8)	0.52^a^

Abbreviation: SD, Standard deviation.

^a^ Analysis of Variance (ANOVA) test.

**FIGURE 2:**
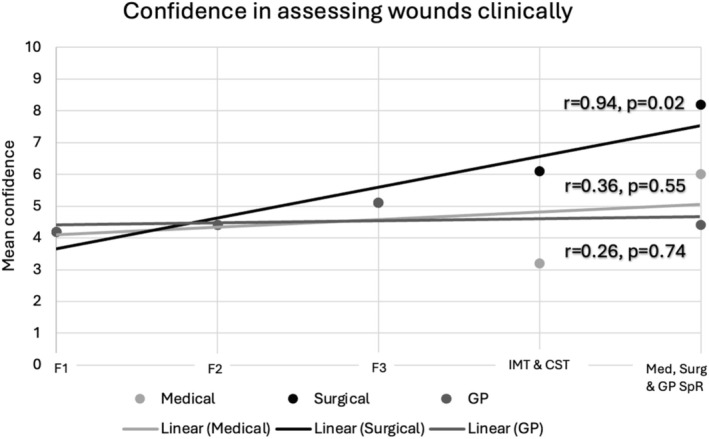
Showing confidence in assessing wounds clinically with line of best fit.

**FIGURE 3:**
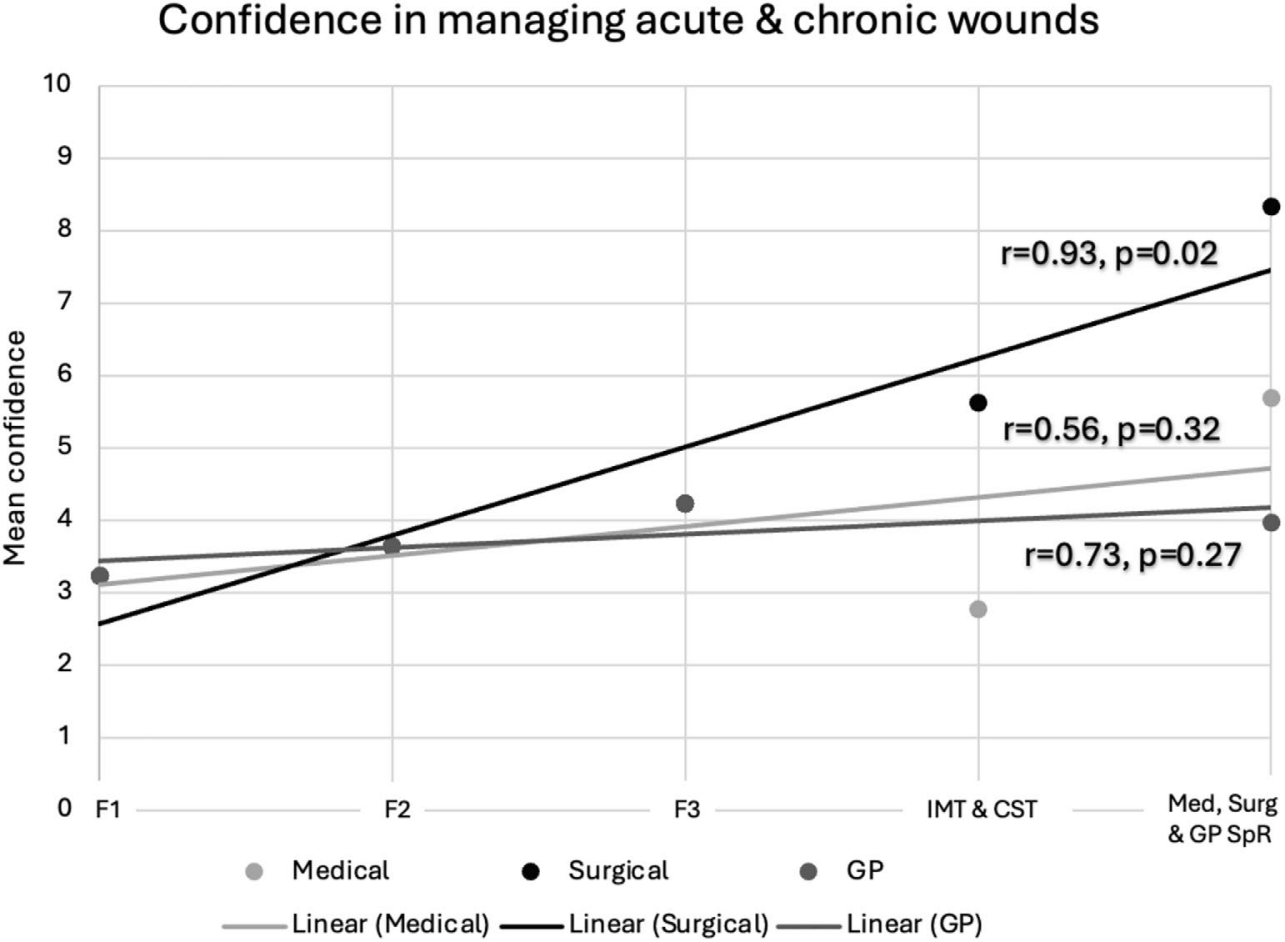
Showing confidence in managing acute and chronic wounds with line of best fit.

**FIGURE 4:**
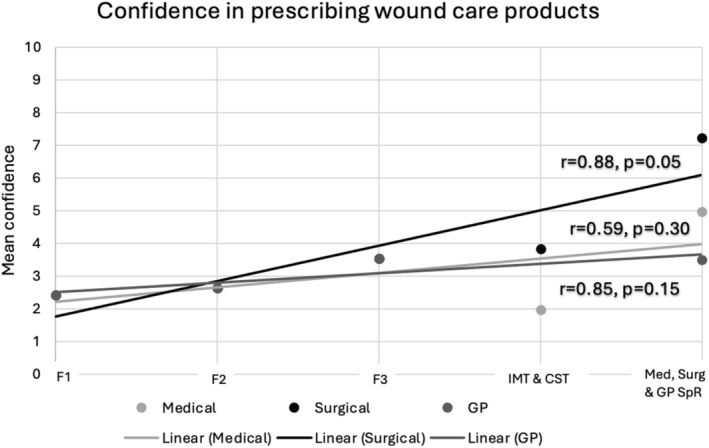
Showing confidence in prescribing wound care products with line of best fit.

When performing further analysis with regards to the relationship between confidence in assessing wounds clinically and seniority in training, there was a statistically significant relationship only among surgical trainees (*p* = 0.01) with a Pearson coefficient of 0.94. The same relationship was observed when looking at confidence in managing acute and chronic wounds and prescribing wound care products. This shows that confidence significantly improved in those domains as surgical trainees progressed from foundation trainees to core surgical trainees to surgical registrars. This difference was not observed, however, as medical or GP trainees progressed from foundation trainees to IMT to medical registrars or in GP trainees transitioning from foundation trainees to GP registrars. The results of this analysis are displayed in Figures 2, 3, 4 in the form of a scatter plot with a line of best fit.

## 8 Discussion

Our study shows that, despite being a part of the core curriculum in 81% of medical schools, only 65% of doctors in training received basic teaching on wound healing during medical school. This finding is concordant with a previous study which found that 68% of medical students receiving teaching on wound healing [8]. Given there is limited further education, with only 18% of foundation trainees and 25% of postgraduate trainees receiving further teaching on wound healing, it is unsurprising that there is poor trainee confidence when managing wounds. The only reliable teaching doctors in training appear to receive on wound healing is during medical school; nevertheless, over a third of doctors have not received any teaching at all on the topic. Furthermore, those participants who did receive teaching on wound healing at medical school thought it was inadequate, and that they would benefit from further teaching.

The most popular method of delivering teaching on wound education in medical schools was through lectures alone, reported in 75% of cases. Similar results were found in previous studies carried out amongst medical students. However, when asked about their preferred method of teaching, lectures were the least popular option. Our study suggests how we can overcome the paucity of physician knowledge. Participants indicate that they would prefer to learn through hands‐on teaching, either in a clinical setting or a simulation. To meet GMC guidance stating that a newly qualified doctor should be able to take wound swabs and carry out appropriate wound closure upon qualifying from medical school, lectures alone would not allow students to develop those clinical competencies [14]. This underscores the need for restructuring wound education within medical schools to meet both the GMC's and trainees' standards.

While reassuring to see that surgical trainees felt confident in assessing and managing wounds along with prescribing wound care products, the confidence in such tasks was low amongst all other trainee groups. All trainee groups thought that wound care was integral to their future clinical practice, reinforced by a demand for further teaching on wound healing irrespective of speciality or grade. While certain surgical specialities deal with more advanced forms of wound care, such as Plastic, Orthopaedic and Oral and Maxillofacial surgery, all doctors should be able to provide basic wound care [15]. This knowledge will help identify and prevent the worsening of wounds, identify when specialist referral is required or give patients basic wound care advice [16]. Moreover, GPs are increasingly the first point of call for wound management as the NHS aims for 85% of elective procedures to be day cases [5]. Therefore, it is important that wound education is improved across specialities to improve patient care.

When comparing the relationship between seniority in training and confidence in assessing and managing wounds and prescribing wound care products, there was a significant difference only amongst surgical trainees. For medical and GP trainees, the confidence level in those tasks remained similar to those of foundation trainees and did not correlate with seniority. This shows that surgical training is adequately providing wound education to doctors as they progress from core surgical training to the registrar level. Both an increased clinical exposure to wounds and the requirement to pass the MRCS exam likely contribute to improved wound healing knowledge in the surgical cohort when compared to medical and GP trainees. The MRCS exam contains aspects of wound healing and its management as reported by participants in our questionnaire. The Membership of the Royal College of Physicians (MRCP) and Membership of the Royal College of General Practitioners (MRCGP) exams have a minimal focus on wound management, and may further explain the low confidence of trainees in this topic [17, 18].

In this study, we employed a mixed methods approach to distribute our questionnaire, aiming to gather responses from a broad spectrum of medical practitioners across the UK. However, it is important to note that our respondents did not represent a random sample of doctors nationwide. Response rates were notably higher in regions where the authors are based (Wales, the Northwest and the Oxford deaneries), likely due to increased motivation amongst participants from these areas. Foundation doctors were well represented and had a broader geographic distribution compared to other groups. Consequently, caution should be taken when generalising these findings to the overall population of UK physicians. This study also carried the limitations inherent in any self‐reported survey. Responses are susceptible to social bias. For example, healthcare professionals, aware of the expectation to follow official guidelines, may have reported higher adherence rates than were practised.

A key strength of this survey is its large and diverse respondent pool, which includes various practice settings, specialties and levels of training. This diversity provides a more comprehensive view of the overall knowledge base in the field. Additionally, our study is the first to examine the confidence levels of training doctors in assessing and managing wounds. Confidence has been used as a proxy for clinical skill in the literature, correlating with competence across assessed domains despite its non‐specific nature [19, 20]. It remains the only cross‐sectional study, at the time of publishing, to compare confidence in wound management across specialties and training pathways. The CHERRIES protocol was implemented to minimise sources of bias in questionnaire design and distribution; however, recall bias amongst participants was difficult to fully eliminate.

Although this study shows that doctors' knowledge of wound care appears somewhat limited, established literature does not indicate the same gap for nursing colleagues [21]. This discrepancy likely reflects the current educational focus, as most wound care teaching initiatives are directed toward nursing and allied health professionals, who manage wound care more routinely in clinical settings. Near‐peer learning, a well‐established, cost‐effective and practical teaching approach, could be beneficial here [22]. Senior nurses, for instance, could be engaged to provide wound care training for doctors, helping to bridge the knowledge gap in a clinical context.

Finally, this research aims to justify and support the need for further studies investigating the impact of poor wound care knowledge of clinicians on patient outcomes. Alongside future work assessing in vivo wound management skills, key learning objectives may be identified to guide wound care education and thus improve patient care.

## 9 Conclusion

Overall, our study has shown that insufficient training and sub‐optimal methods of teaching have led to reduced confidence amongst trainees in managing wounds. Surgical trainees are more confident than their medical counterparts in managing wounds, and confidence in wound management was only linked to seniority amongst surgical trainees. Further education should be provided at all levels of training, ideally through hands‐on clinical practice either in a clinical setting or simulation.

## Ethics Statement

This study did not require ethical approval because of fulfilling the local health authority's policy for the quality improvement project. Furthermore, this study involved only the use of a non‐sensitive, completely anonymous educational survey; those surveyed did so voluntarily and were doctors in training, considered non‐vulnerable participants, and participation did not induce any psychological stress or anxiety.

## Conflicts of Interest

The authors declare no conflicts of interest.

## Data Availability

The data that support the findings of this study are not openly available however anonymised data can be made available upon reasonable request.
